# Survival Outcomes According to Adjuvant Treatment and Prognostic Factors Including Host Immune Markers in Patients with Curatively Resected Ampulla of Vater Cancer

**DOI:** 10.1371/journal.pone.0151406

**Published:** 2016-03-14

**Authors:** Hye rim Ha, Do-Youn Oh, Tae-Yong Kim, KyoungBun Lee, Kyubo Kim, Kyung-Hun Lee, Sae-Won Han, Eui Kyu Chie, Jin-Young Jang, Seock-Ah Im, Tae-You Kim, Sun-Whe Kim, Yung-Jue Bang

**Affiliations:** 1 Department of Internal medicine, Seoul National University Hospital, Seoul, South Korea; 2 Cancer Research Institute, Seoul National University College of Medicine, Seoul, South Korea; 3 Department of Pathology, Seoul National University Hospital, Seoul, South Korea; 4 Department of Radiation Oncology, Seoul National University Hospital, Seoul, South Korea; 5 Department of Surgery, Seoul National University Hospital, Seoul, South Korea; University General Hospital of Heraklion and Laboratory of Tumor Cell Biology, School of Medicine, University of Crete, GREECE

## Abstract

**Background:**

Ampulla of Vater cancer (AoV Ca) is a rare tumor, and its adjuvant treatment has not been established. The purpose of this study was to find out prognostic factors including host immunity and role of adjuvant treatment in AoV Ca.

**Methods and Findings:**

We reviewed 227 AoV Ca patients with curative resection. Clinical characteristics, adjuvant treatment, disease-free survival (DFS) and overall survival (OS) were analyzed. Among all patients, 63.9, 36.1 and 33.9% had T1/T2, T3/T4 stage and lymph node-positive disease (LN+), respectively. OS of all patients was 90.9 months (95% CI: 52.9–129.0). OS was different according to neutrophil-to-lymphocyte ratio (HR 1.651, 95% CI: 1.11–2.47), platelet-to-lymphocyte ratio (HR 1.488, 95% CI: 1.00–2.21) and systemic inflammatory index (HR 1.669, 95% CI: 1.13–2.47). In multivariate analysis, adverse prognostic factors for OS included vascular invasion (HR 2.571, 95% CI: 1.20–5.53) and elevated CA 19–9 (HR 1.794, 95% CI: 1.07–3.05). A total of 104 patients (46.3%) received adjuvant treatment (25 out of 111of T1/T2 & LN (-), 79 out of 116 of T3/T4 or LN (+)). In T3/T4 or LN (+) stage, adjuvant CCRT with maintenance chemotherapy provided the longest OS (5-year OS rate: 47.0 *vs*. 41.4%).

**Conclusions:**

Vascular invasion and elevated CA 19–9 were adverse prognostic factors in resected AoV Ca. In T3/T4 or LN (+) stage, adjuvant CCRT with maintenance chemotherapy provided the best survival outcome. Adjuvant treatment should be further defined in AoV Ca, especially with poor prognostic factors.

## Introduction

The annual incidence of biliary tract cancer (BTC) in the Western world is about 5–6 per 100,000, while the annual incidence in Korea is 10 per 100,000.[1, 2] BTC has a worse prognosis than other malignancies.[2] Surgical resection is the only treatment modality which offers a chance of cure.[3] Approximately 40 ~ 50% of cholangiocarcinoma and 30% of gallbladder cancer patients undergo surgery; however, even in those resected cases, many patients experience cancer recurrence.[4, 5] In 1999, there was a randomized controlled study to evaluate the role of adjuvant concurrent chemoradiotherapy (CCRT) in pancreatic and biliary cancers by The European Organization for Research and Treatment of Cancer, which failed to show survival gain.[6] Other retrospective studies of the role of radiotherapy after surgical resection showed better 5-year loco-regional disease-free survival (DFS) and overall survival (OS) rates, and several retrospective analyses also showed significantly better survival outcomes in lymph node-positive patients on adjuvant CCRT.[7–9]

Ampulla of Vater cancer (AoV Ca) accounts for 10–15% of BTC in Korea, which arises from distal to the confluence of the common bile duct with the main pancreatic duct.[10] Initial presentations of AoV Ca are usually related to biliary obstruction such as jaundice, red urine and pruritus, potentially resulting in early detection.[11] Approximately 80% of AoV Ca patients were detected at a potentially resectable stage at the time of diagnosis. [12] Prognosis of AoV Ca has been favorable compared with other biliary tract cancers originating from the intrahepatic or extrahepatic bile duct or gallbladder. However, resected patients relapse in many cases, which leads to an eventual 5-year survival rate of 20~50%.[7, 13] The identification of patients with poor prognosis after curative resection is important to improve survival outcomes. In parallel, the role of adjuvant treatment should be accurately defined in patients with poor prognosis. Because of the relatively low incidence of AoV Ca, a prospective study design is extremely difficult to answer those questions.

Several studies have reported on the prognostic factors of AoV Ca. Nowadays, host immunity and peritumoral inflammation are considered important factors in the carcinogenesis and prognosis of solid tumors. [14–17] However, in BTC, including AoV Ca, the prognostic role of host immunity and peritumoral inflammation has not been well documented.

In this study, we evaluated the prognostic factors to define the AoV Ca patients with poor prognosis after curative resection. In this analysis, we included immunity/inflammation markers. The other important purpose of this study was to determine the role of adjuvant treatment in AoV Ca.

## Methods

### Patients and data collection

This study was a retrospective analysis of de-identified patient-level data from medical charts. Patients who were diagnosed with AoV Ca and who underwent curative resection at the Seoul National University Hospital between 1997 and 2012 were enrolled. Diagnosis was confirmed by tissue pathology. Data of baseline demographics were collected, including gender, ECOG, stage, laboratory tests (total bilirubin, albumin, carcinoembryonic antigen (CEA), carbohydrate antigen 19–9 (CA 19–9), and neutrophil, platelet and lymphocyte counts). The data of adjuvant treatment patterns were also collected, including chemotherapy, radiotherapy and CCRT. Survival outcomes including disease-free survival (DFS) and overall survival (OS) were obtained as well.

### Statistical analysis

Statistical analysis of categorical variables was performed using Pearson’s chi-square test or Fisher’s exact test, as appropriate. A t-test was used for comparison of means. Median DFS and OS for all patients were calculated using the Kaplan-Meier method and comparisons between groups were made using log-rank tests.

Neutrophil, lymphocyte and platelet count were obtained from preoperative laboratory tests. We calculated neutrophil-to-lymphocyte ratio (NLR) and platelet-to-lymphocyte ratio (PLR) as neutrophil and platelet counts divided by lymphocyte counts, respectively. We also used the systemic inflammatory index (SII) which was determined as neutrophil x platelet/ lymphocyte.[18] The cut-off values for NLR, PLR and SII were obtained using receiver operating characteristic (ROC) curve analysis for predicting OS.

The impact of continuous numerical variables on clinical outcomes was evaluated using Cox regression. Multivariate analysis for DFS and OS was also performed using Cox regression models. Factors with *p*<0.05 in univariate analysis were examined in multivariate regression models. All statistical tests were two-sided, with significance defined as *p*<0.05.

### Ethics

The study protocol was reviewed and approved by the Institutional Review Board of Seoul National University Hospital (H-1306-109-500). All studies were conducted according to guidelines for biomedical research (Declaration of Helsinki). Written informed consent was not given by participants but patients’ record and information was anonymized and de-identified prior to analysis.

## Results

### Patient characteristics

A total of 227 patients were included in this analysis (Table 1). Median age was 61.5 years old (range: 33.8–88.2), and there were 125 male patients (55.1%). With regard to T stage, T1/T2 was found in 63.9% of patients, and 77 patients (33.9%) had lymph node (LN) involvement. Stage I A/B and stage II A/B according to the American Joint Committee on Cancer Staging system, seventh edition, were shown in 58/53 and 38/73 patients, respectively. A total of 216 patients had adenocarcinoma on pathology review. Twenty-two patients (9.7%) had poorly differentiated histology. Mean (median, 95% CI) value of NLR was 2.32 (1.92, 0.39–20.50). Mean (median, 95% CI) value of PLR was 179.2 (158.8, 11.7–692.3). Mean (median, 95% CI) value of SII was 709.8 (544.8, 86.5–6478.0).

**Table 1:** Patient characteristics.

		Number	Percent (%)
Age			
	Median(range)	61.5 (33.8–88.2)	
Sex			
	Male	125	55.1
	Female	102	44.9
T stage			
	T1	68	30
	T2	77	33.9
	T3	77	33.9
	T4	5	2.2
N stage			
	N0	150	66.1
	N1	77	33.9
Stage			
	IA	58	25.6
	IB	53	23.3
	IIA	38	16.7
	IIB	73	32.2
	III	5	2.2
Pathology			
	Adenocarcinoma	216	95.2
	Adenosquamous	2	0.9
	Mucinous	3	1.3
	Neuroendocrine(Gr1,2/G3)	2/2	1.8
	Papillary	2	0.9
Differentiation			
	Well-differentiated	75	33.0
	Moderately-differentiated	124	54.6
	Poorly-differentiated	22	9.7
	Unknown	6	2.6
Lymphatic invasion			
	No	120	52.9
	Yes	76	33.5
	Unknown	31	13.7
Vascular invasion			
	No	177	78.0
	Yes	20	8.8
	Unknown	30	13.2
Perineural invasion			
	No	156	68.7
	Yes	44	19.4
	Unknown	27	11.9
Total bilirubin			
	Normal	110	48.5
	Elevated	112	49.3
	Unknown	5	2.2
Albumin			
	Decreased	42	18.5
	Normal	180	79.3
	Unknown	5	2.2
CEA			
	Normal	202	89.0
	Elevated	14	6.2
	Unknown	11	4.8
CA-19-9			
	Normal	142	62.6
	Elevated	76	33.5
	Unknown	9	4.0
NLR			
	≤ 1.78	100	44.8
	> 1.78	123	54.2
	Unknown	4	1.8
PLR			
	≤192.0	148	65.2
	> 192.0	75	33.0
	Unknown	4	1.8
SII			
	≤ 780.0	146	64.3
	> 780.0	77	33.9
	Unknown	4	1.8

CEA, carcinoembryonic antigen; CA-19-9, carbohydrate antigen-19-9; NLR, neutrophil-to-lymphocyte ratio; PLR, platelet-to-neutrophil ratio; SII, systemic inflammatory index.

**Fig 1:**
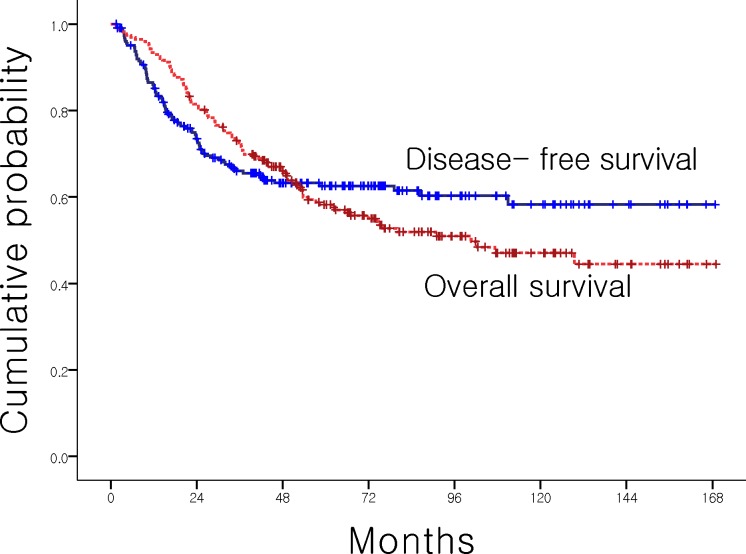
Survival outcomes of AoV Ca patients. Five-year OS rates was 58.2% and DFS rates was 62.5%.

The follow-up duration of all patients was 48.0 months (95% CI: 43.5–52.4). Eighty-two patients experienced relapse and 105 patients were dead at the time of analysis. Median OS was 90.96 months (95% CI: 53.84–128.09), with 5-year OS rate of 58.3%. Median DFS was not reached and 5-year DFS rate was 62.5% (Fig 1).

The cut-off values of NLR, PLR and SII for predicting OS were 1.78, 192.0, and 780.0, respectively. The numbers of patients with NLR, PLR and SII values lower than cut-off were 100 (44.8%), 148 (65.2%) and 146 (64.3%), respectively (Table 1).

### Prognostic factor and clinical outcomes

**Fig 2:**
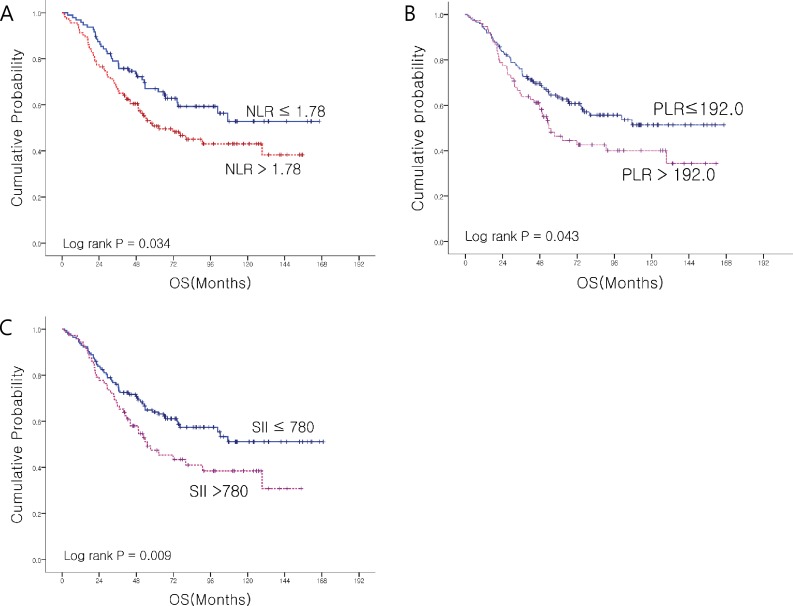
**OS according to NLR (A), PLR (B) & SII(C).** (A)(B) show OS according to NLR and PLR. High NLR and high PNR has poorer OS than low NLR, low PLR. (C) shows OS according to SII, high SII also poorer OS, also.

In univariate analysis, aged <60, CEA, CA-19-9, total bilirubin, NLR, PLR, SII and T/N stage were significant prognostic factors for 5-year OS (Table 2). Patients with lower NLR showed longer survival than patients with higher NLR (not achieved *vs*. 58.2 months, HR 1.651 (95% CI: 1.11–2.47), *p* = 0.012) (Fig 2A). In a similar way, lower PLR was associated with better survival (not achieved *vs*. 49.3 months, HR 1.767 (95% CI: 1.18–2.65), *p* = 0.043) (Fig 2B). Patients with lower SII showed better survival (not achieved *vs*. 53.6 months, HR 1.669 (95% CI: 1.13–2.47), p = 0.010) (Fig 2C). Patient characteristics according to NLR (low *vs*. high) were compared (Table 3). In the higher NLR group, a higher proportion of T3/4 stage, stage II/III, lymphatic/perineural invasion, high PLR, high SII was observed.

**Table 2:** Analysis of prognostic factor for OS.

		5Y- OS (%)	Univariate analysis		Multivariate analysis	
			HR(95% CI)	P	HR(95% CI)	P
Age			1.659 (1.11–2.49)	0.015	1.503 (0.92–2.46)	0.104
	< 60	66.5				
	≥60	52.3				
Size			0.732 (0.49–1.09)	0.121		
	< 2 Cm	54.5				
	≥2 Cm	61.2				
Pathology			1.018 (0.72–1.45)	0.920		
	Adenocarcinoma	58.7				
	Adenosquamous	0.0				
	Mucinous	66.7				
	Neuroendocrine	75.0				
	Papillary	0.0				
Differentiation			1.778 (1.30–2.43)	< 0.001	1.526 (0.98–2.39)	0.064
	Well-diff	76.7				
	Mod-diff	53.9				
	Poorly-diff	27.1				
Lymphatic invasion			1.888 (1.23–2.90)	0.004	0.749 (0.41–1.35)	0.339
	No	72.2				
	Yes	44.1				
Vascular invasion			3.605 (2.11–6.17)	< 0.001	2.616 (1.21–5.67)	**0.015**
	No	65.1				
	Yes	24.0				
Perineural invasion			2.852 (1.83–4.43)	< 0.001	1.549 (0.83–2.87)	0.166
	No	68.2				
	Yes	29.6				
CEA			2.871 (1.57–2.26)	< 0.001	1.473 (0.65–3.36)	0.357
	Normal	60.9				
	Elevated	25.7				
CA-19-9			1.912 (1.29–2.84)	0.001	1.787 (1.06–3.02)	**0.030**
	Normal	67.7				
	Elevated	43.1				
Albumin			0.645 (0.41–1.01)	0.057		
	Decreased	41.0				
	Normal	62.3				
Total bilirubin			2.024 (1.36–3.02)	< 0.001	1.115 (0.65–1.92)	0.695
	Normal	72.9				
	Elevated	44.4				
T stage			1.702 (1.34–2.16)	< 0.001	1.342 (0.96–1.88)	0.085
	T1	74.7				
	T2	66.1				
	T3	38.9				
	T4	0.0				
N stage			2.641 (1.80–3.88)	< 0.001	1.617 (0.93–2.80)	0.086
	N0	69.7				
	N1	36.2				
NLR			1.651 (1.11–2.47)	0.012	1.280 (0.70–2.33)	0.418
	≤1.78	68.3				
	>1.78	49.4				
PLR			1.488 (1.00–2.21)	0.043	0.686 (0.35–1.34)	0.268
	≤192.0	63.8				
	>192.0	46.4				
SII			1.669 (1.13–2.47)	0.010	0.924 (0.44–1.93)	0.833
	≤780	64.7				
	>780	45.1				

NA, not achieved; CEA, carcinoembryonic antigen; CA-19-9, carbohydrate antigen-19-9; NLR, neutrophil-to-lymphocyte ratio; PLR, platelet-to-neutrophil ratio; SII, systemic inflammatory index.

**Table 3:** Comparison of patient characteristics according to NLR.

		NLR≤1.78[N (%)]	NLR>1.78[N (%)]	P value
Age				
	Median(range)	61.4(37.0–88.2)	62.0(33.8–86.0)	0.993
Sex				
	Male	50(50.0)	74(60.1)	0.129
	Female	50(50%)	49(39.8)	
T stage				
	T1	28(28.0)	40(32.5)	**0.021**
	T2	43(43.0)	32(14.3)	
	T3	29(29.0)	47(38.2)	
	T4	0(0.0)	4(3.3)	
N stage				
	N0	72(72.0)	77(62.6)	0.138
	N1	28(28.0)	46(37.4)	
Stage				
	IA	25(25.0)	33(26.8)	**0.048**
	IB	32(32.0)	21(17.1)	
	IIA	15(15.0)	23(18.7)	
	IIB	28(28.0)	42(34.1)	
	III	0(0.0)	4(3.3)	
Pathology				
	Adenocarcinoma	97(97.0)	115(93.5)	0.338
	Adenosquamous	0(0.0)	2(1.6)	
	Mucinous	2(2.0)	1(0.8)	
	Neuroendocrine	1(1.0)	3(2.4)	
	Papillary	0(0.0)	2(1.6)	
Differentiation				
	Well-differentiated	35(35.3)	38(31.9)	0.392
	Moderately-differentiated	57(57.6)	66(55.5)	
	Poorly-differentiated	7(7.0)	15(12.6)	
Lymphatic invasion				
	No	61(68.5)	58(54.2)	**0.041**
	Yes	28(31.5)	49(45.8)	
Vascular invasion				
	No	84(94.4)	92(86.0)	0.053
	Yes	5(5.6)	15(14.0)	
Perineural invasion				
	No	76(84.4)	79(72.5)	**0.043**
	Yes	14(15.6)	30(27.5)	
Total bilirubin				
	Normal	51(52.6)	58(47.2)	0.424
	Elevated	46(47.4)	65(52.8)	
Albumin				
	Decreased	16(52.6)	26(21.1)	0.384
	Normal	81(83.5)	97(78.9)	
CEA				
	Normal	92(96.8)	109(91.6)	0.110
	Elevated	3(3.2)	10(8.4)	
CA-19-9				
	Normal	68(72.3)	74(60.7)	0.073
	Elevated	26(27.7)	48(39.3)	
PLR				
	≤192.0	85(85.0)	63(51.2)	**<0.001**
	> 192.0	15(15.0)	60(48.8)	
SII				
	≤ 780	95(95.0)	51(41.5)	**<0.001**
	> 780	5(5.0)	72(58.5)	

CEA, carcinoembryonic antigen; CA-19-9, carbohydrate antigen-19-9; NLR, neutrophil-to-lymphocyte ratio; PLR, platelet-to-neutrophil ratio; SII, systemic inflammatory index.

Regarding pathologic findings, degree of differentiation and lymphatic/vascular/perineural invasion were also significant prognostic factors for OS. On multivariate analysis, vascular invasion and elevated CA 19–9 were significant poor prognostic factors for 5-year OS (Table 2).

Adverse prognostic factors for 5-year DFS were differentiation, lymphatic/vascular/perineural invasion, CEA, CA 19–9, total bilirubin and T/N stage on univariate analysis. Differentiation and T/N stage showed significant differences for DFS on multivariate analysis (S1 Table).

### The patterns of adjuvant treatment

After curative resection of tumor, 104 patients (45.8%) received adjuvant treatment. Adjuvant treatment modalities according to tumor stage are shown in S2 Table.

A total 59 patients received adjuvant CCRT with maintenance chemotherapy, and 32 patients received adjuvant CCRT. Eight and five patients received adjuvant chemotherapy only and adjuvant radiotherapy only, respectively. The most commonly used chemotherapy was 5-FU based one. During CCRT, the regimen 5-FU 500 mg/m^2^, D1,2,3 q 4 weeks was most commonly used, followed by 5-FU/leucovorin (375 mg/m^2^, 20 mg/m^2^, respectively, D1-5, q 4 weeks). During maintenance chemotherapy or adjuvant chemotherapy alone, 5-FU 500 mg/m^2^, D1-5 q 4 weeks was most commonly used for 6 months. Radiotherapy was administered at a dose of 45 Gy in 25 fractions.

**Fig 3:**
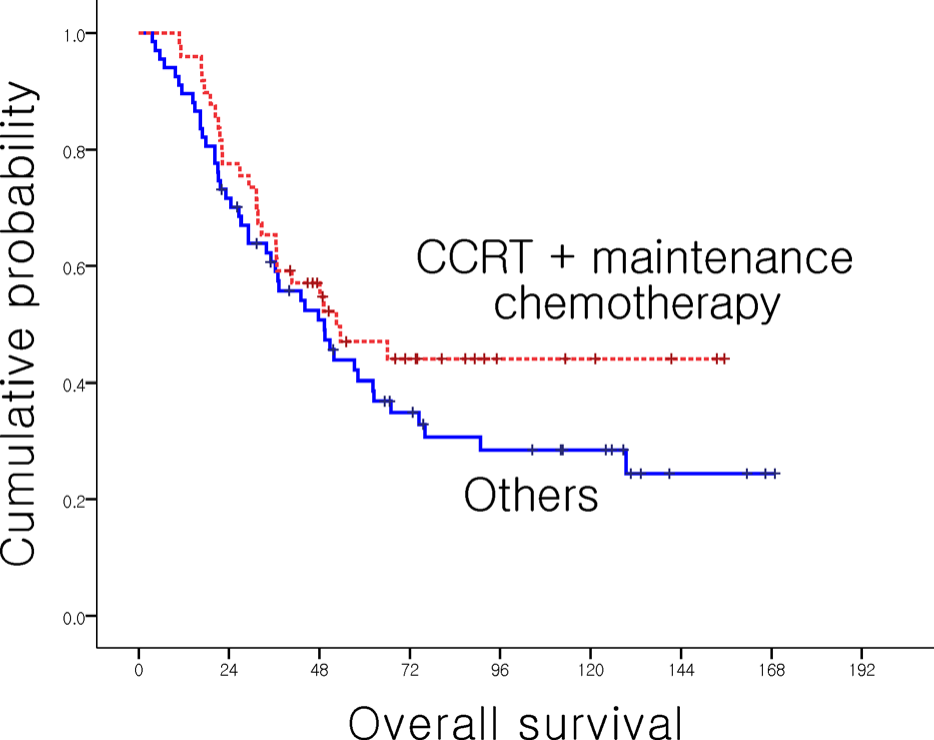
OS of AoV Ca in T3/T4 or LN (+). CCRT with maintenance provided improved OS than no adjuvant treatment in T3/T4 or LN (+).

When we analyzed survival outcomes according to adjuvant treatment, there was no significant difference in stage 1A and 1B. However, in T3/T4 or LN (+) stage, the patients who received adjuvant CCRT with maintenance chemotherapy had better 5-year OS, even though the finding was not statistically significant (Table 4, Fig 3).

**Table 4:** Treatment outcomes by stage & adjuvant treatment.

		5-Y DFS (%)	Log-rank P	5-Y OS (%)	Log-rank P
Total					
	CCRT + maintenance chemotherapy	52.1	0.022	52.0	0.336
	CCRT	45.4		46.5	
	Chemotherapy	62.2		48.6	
	Radiotherapy	60.0		26.7	
	No treatment	72.9		66.0	
T1/T2 &LN(-)					
	CCRT + maintenance chemotherapy	75.0	0.165	77.8	0.699
	CCRT	53.6		71.4	
	Chemotherapy	75.0		55.6	
	Radiotherapy	66.7		33.3	
	No treatment	86.5		76.5	
T3/T4 or LN(+)					
	CCRT + maintenance chemotherapy	**47.9**	0.844	**47.0**	0.730
	CCRT	43.1		41.3	
	Chemotherapy	33.3		33.3	
	Radiotherapy	50.0		0.0	
	No treatment	**43.5**		**41.4**	

CCRT; concurrent chemoradiotherapy, LN; lymph node, DFS; disease-free survival, OS; overall survival

In patients who received adjuvant treatment, NLR, PLR and SII were all important factors for OS. However, this was not the case in patients without adjuvant treatment (Table A and B in S1 File).

## Discussion

In this study, we found that in curatively resected AoV Ca, vascular invasion in pathologic examination and elevated CA 19–9 were poor prognostic factors. Patients who had T3/T4 or LN (+) tumors showed good survival when they received adjuvant CCRT with maintenance chemotherapy.

Tumor stage, lymph node involvement and vascular/perineural invasion were well-known prognostic factors in biliary tract cancer.[19] In our study, T/N stage, presence of lymphatic/vascular/perineural invasion, histologic differentiation and elevated total bilirubin/CEA/CA 19–9 were adverse prognostic factors.

In cancer development and progression, the role of inflammation has been highlighted.[15–17, 20] As systemic inflammatory response is activated, neutrophils increase, and in parallel, lymphocytes decrease in peripheral blood. For several years, the index representing the systemic inflammatory state has been developed and several markers such as NLR, PLR and SII have been analyzed in various tumor conditions except AoV Ca.[21, 22] Tumor antigens elicit an adaptive immune response by inflammatory cells, macrophages and lymphocytes. CD4+ T cells and CD8+ T cells have important roles in this process, and especially tumor-infiltrating CD8+ T lymphocytes improve prognosis in several cancers.[23, 24] NLR, PLR and SII may represent these immune response processes and be of prognostic significance.[25, 26]

We analyzed the association of OS and host immunity and inflammation status such as NLR, PLR and SII. The patients with NLR ≤1.78 or PLR ≤192.0 or SII ≤780.0 showed significantly prolonged OS. We selected the cut-off values of NLR, PLR and SII using ROC analysis for OS. NLR and PLR showed a linear relationship (r^2^ = 0.82) and NLR and SII also showed a linear relationship (r^2^ = 0.88). Patients with higher NLR included a higher proportion of T3/4 stage, stage II/III and lymphatic/perineural invasion compared with patients with lower NLR. To the best of our knowledge, this is the most extensive analysis in AoV Ca patients focused on host immunity and inflammation status.

The role of adjuvant treatment in BTC patient has not been established. An earlier retrospective analysis of survival outcomes in patients with adjuvant therapy showed that OS was improved insignificantly.[27] Recently, another study also reported that neoadjuvant and adjuvant chemotherapy did not provide survival benefit.[28] However, a meta-analysis reported survival benefit in patients with LN (+) or R1 resection by adjuvant therapy.[29] The study was reported that the patients with KRAS^G12D^ mutation show poor prognoses and high risk of early recurrence, and adjuvant therapy will be effective in the high risk patients.[30] In these circumstances, according to National Comprehensive Cancer Network (NCCN) guidelines, adjuvant therapy is recommended for R1 or R2 resected or LN (+) patients. In case of R0 resection with no LN involvement or carcinoma in situ at resection margins, four options are all recommended, that is, observation or fluoropyrimidine-based chemoradiation or fluoropyrimidine-based or gemcitabine- based chemotherapy or clinical trial.

In our study, 25% of patients received adjuvant treatment in T1/T2 & LN (-) stage. The percentage of delivered adjuvant treatment was increased with stage, where nearly 70% of patients with T3/T4 or LN (+) stage received adjuvant treatment. Because LN involvement is a well-known adverse prognostic factor, most patients with LN (+) or T3/T4 tumors received adjuvant treatment. CCRT followed by maintenance chemotherapy, mostly 5-FU-based, was the most commonly used adjuvant treatment modality in our study. These data gave us information on the adjuvant treatment regimens for AoV Ca. While adjuvant treatment did not provide survival benefit in T1/T2 stage patients, adjuvant CCRT with maintenance chemotherapy resulted in better survival in T3/T4 or LN-positive patients (no treatment vs. CCRT with maintenance chemotherapy; 41.4 *vs*. 47.0%, *p* = 0.182). Although it was not statistically significant, it suggested the potential benefit of CCRT with maintenance chemotherapy in this population. Adjuvant chemotherapy, adjuvant radiotherapy and adjuvant CCRT without maintenance chemotherapy did not have an impact on the survival of T3/T4 or LN (+) patients as well as those T1/T2 & LN (-).

One of the limitations of our study was the design, i.e., retrospective, single center study. The adjuvant treatment was not applied based on a consistent principle of guidelines, and therefore, the proportion of adjuvant treatment was different according to clinical factors such as stage. It was very difficult to see the genuine impact on prognosis of clinical factors and adjuvant treatment. Other limitation is relatively short follow-up duration, even though eighty-two patients experienced relapse and 105 patients were dead at the time of analysis. This relative short follow-up time might mask the survival difference that occurs later in the time course.

Nonetheless, our study has a value of providing information on adverse prognostic factors including host immunity and inflammation status and clinical outcomes of adjuvant treatment modalities in a relatively large AoV Ca cohort.

In conclusion, the AoV Ca patients with vascular invasion and elevated CA 19–9 showed poor prognosis after curative resection. Host immunity and inflammation status represented by NLR, PLR or SII were also important for the prognosis. In T3/4 or LN-positive stage, patients who received adjuvant CCRT with maintenance chemotherapy showed favorable survival. Adjuvant treatment should be further defined in AoV Ca, especially with poor prognostic factors.

## Supporting Information

S1 File Analysis of prognostic factors for OS according to adjuvant treatment. (DOCX)

S1 Table Analysis of prognostic factor for DFS. (DOCX)

S2 Table The patterns of adjuvant treatment. (DOCX)
