# Supplementary material for: Survival Outcomes According to Adjuvant Treatment and Prognostic Factors Including Host Immune Markers in Patients with Curatively Resected Ampulla of Vater Cancer
Source: PLoS One. 2016 Mar 14;11(3):e0151406. doi: 10.1371/journal.pone.0151406 (PMC4790941; doi:10.1371/journal.pone.0151406)
Supplement: S1 File — (DOCX) [file pone.0151406.s001.docx]

**S1. Analysis of prognostic factors for OS according to adjuvant treatment**

1. **Patients without adjuvant treatment**

|  | Univariate analysis | | Multivariate analysis | |
| --- | --- | --- | --- | --- |
|  | HR(95% CI) | P | HR(95% CI) | P |
| Age | 3.827 (1.72- 8.53) | **0.001** | 4.128 (1.41- 12.11) | **0.010** |
| Size | 0.521 (0.29- 0.93) | **0.028** | 0.346 (0.14- 12.11) | **0.019** |
| Pathology | 0.853 (0.49- 1.50) | 0.576 |  |  |
| Differentiation | 2.450 (1.51- 3.96) | **< 0.001** | 1.070 (0.48- 2.39) | 0.870 |
| Lymphatic invasion | 1.414 (0.70- 2.85) | 0.333 |  |  |
| Vascular invasion | 2.913 (1.18- 7.21) | **0.021** | 2.063 (0.50- 8.48) | 0.315 |
| Perineural invasion | 4.402 (2.23- 8.68) | **< 0.001** | 1.367 (0.40- 4.70) | 0.620 |
| CEA | 1.668 (0.60- 4.65) | 0.329 |  |  |
| CA-19-9 | 2.402 (1.36- 4.24) | **0.002** | 1.659 (0.77- 3.60) | 0.200 |
| Albumin | 0.750 (0.40- 1.42) | 0.375 |  |  |
| Total bilirubin | 2.139 (1.21- 3.79) | **0.009** | 0.708 (0.32- 1.59) | 0.402 |
| T stage | 1.797 (1.27- 2.55) | **< 0.001** | 1.879 (0.98- 3.62) | 0.059 |
| N stage | 3.644 (2.06- 6.44) | **< 0.001** | 1.848 (0.71- 4.78) | 0.205 |
| NLR | 1.376 (0.78- 2.43) | 0.269 |  |  |
| PLR | 1.103 (0.60- 2.03) | 0.752 |  |  |
| SII | 1.245 (0.70- 2.20) | 0.451 |  |  |

**(B) Patients with adjuvant treatment**

|  | Univariate analysis | | Multivariate analysis | |
| --- | --- | --- | --- | --- |
|  | HR(95% CI) | P | HR(95% CI) | P |
| Age | 1.160 (0.68- 1.97) | 0.583 |  |  |
| Size | 0.922 (0.53- 1.61) | 0.775 |  |  |
| Pathology | 1.583 (0.93- 2.69) | 0.090 |  |  |
| Differentiation | 1.243 (0.78- 1.98) | 0.357 |  |  |
| Lymphatic invasion | 1.878 (1.02- 3.47) | **0.045** | 1.558 (0.71- 3.41) | 0.266 |
| Vascular invasion | 3.519 (1.78- 6.95) | **< 0.001** | 2.242 (0.88- 5.73) | 0.092 |
| Perineural invasion | 1.793 (1.00- 3.22) | 0.051 |  |  |
| CEA | 5.098 (2.36- 11.03) | **< 0.001** | 4.099 (1.50- 11.18) | **0.006** |
| CA-19-9 | 1.539 (0.88- 2.68) | 0.128 |  |  |
| Albumin | 0.490 (0.26- 0.94) | **0.031** | 0.591 (0.28- 1.23) | 0.161 |
| Total bilirubin | 1.700 (0.96- 3.00) | 0.067 |  |  |
| T stage | 1.533 (1.04- 2.26) | **0.031** | 1.319 (0.92- 2.13) | 0.256 |
| N stage | 1.934 (1.12- 3.34) | **0.018** | 1.301 (0.65- 2.62) | 0.462 |
| NLR | 1.940 (1.09- 3.45) | **0.024** | 0.965 (0.40- 2.32) | 0.967 |
| PLR | 1.780 (1.04- 3.04) | **0.034** | 0.877 (0.37- 2.09) | 0.767 |
| SII | 2.356 (1.38- 4.03) | **0.002** | 2.254 (0.79- 6.41) | 0.127 |

CEA; carcinoembryonic antigen, CA-19-9; carbohydrate antigen-19-9, NLR; neutrophil-to-lymphocyte ratio, PLR; platelet-to-neutrophil ratio, SII; systemic inflammatory index
