# Supplementary material for: Survival Outcomes According to Adjuvant Treatment and Prognostic Factors Including Host Immune Markers in Patients with Curatively Resected Ampulla of Vater Cancer
Source: PLoS One. 2016 Mar 14;11(3):e0151406. doi: 10.1371/journal.pone.0151406 (PMC4790941; doi:10.1371/journal.pone.0151406)
Supplement: S1 Table — (DOCX) [file pone.0151406.s002.docx]

**S1. Analysis of prognostic factor for DFS**

|  |  | 5-Y DFS (%) | Univariate analysis | | Multivariate analysis | |
| --- | --- | --- | --- | --- | --- | --- |
|  |  |  | HR(95% CI) | P | HR(95% CI) | P |
| Age |  |  | 1.164 (0.75- 1.81) | 0.501 |  |  |
|  | < 60 | 63.5 |  |  |  |  |
|  | ≥60 | 61.8 |  |  |  |  |
| Size |  |  | 1.013 (0.62- 1.57) | 0.957 |  |  |
|  | < 2 Cm | 65.4 |  |  |  |  |
|  | ≥2 Cm | 63.0 |  |  |  |  |
| Pathology |  |  | 1.075 (0.77- 1.49) | 0.665 |  |  |
|  | Adenocarcinoma | 63.1 |  |  |  |  |
|  | Adenosquamous | 50.0 |  |  |  |  |
|  | Mucinous | 33.3 |  |  |  |  |
|  | Neuroendocrine | 75.0 |  |  |  |  |
|  | Papillary | 50.0 |  |  |  |  |
| Differentiation |  |  | 2.176 (0.46- 1.58) | < 0.001 | 1.980 (1.19- 3.30) | **0.009** |
|  | Well-diff | 84.6 |  |  |  |  |
|  | Mod-diff | 55.2 |  |  |  |  |
|  | Pooly-diff | 39.7 |  |  |  |  |
| Lymphatic invasion |  |  | 2.919 (1.79- 4.77) | < 0.001 | 0.816 (0.44- 1.52) | 0.521 |
|  | No | 76.2 |  |  |  |  |
|  | Yes | 45.6 |  |  |  |  |
| Vascular invasion |  |  | 2.740 (1.43- 5.25) | 0.002 | 0.846 (0.39- 1.83) | 0.670 |
|  | No | 67.3 |  |  |  |  |
|  | Yes | 40.2 |  |  |  |  |
| Perineural invasion |  |  | 3.080 (1.88- 5.06) | < 0.001 | 0.711 (0.39- 1.29) | 0.262 |
|  | No | 71.8 |  |  |  |  |
|  | Yes | 37.7 |  |  |  |  |
| CEA |  |  | 2.781 (1.38- 5.59) | 0.004 | 1.891 (0.75- 4.75) | 0.176 |
|  | Normal | 64.7 |  |  |  |  |
|  | Elevated | 40.0 |  |  |  |  |
| CA-19-9 |  |  | 1.973 (1.25- 3.10) | 0.003 | 1.432 (0.81- 2.55) | 0.221 |
|  | Normal | 71.2 |  |  |  |  |
|  | Elevated | 51.2 |  |  |  |  |
| Albumin |  |  | 1.506 (0.40- 1.11) | 0.121 |  |  |
|  | Decreased | 52.9 |  |  |  |  |
|  | Normal | 66.0 |  |  |  |  |
| Total bilirubin |  |  | 2.037 (1.28- 3.24) | 0.003 | 1.087 (0.59- 1.99) | 0.788 |
|  | Normal | 75.3 |  |  |  |  |
|  | Elevated | 51.4 |  |  |  |  |
| T stage |  |  | 2.366 (1.76- 3.18) | <0.001 | 1.886 (1.26- 2.83) | **0.002** |
|  | T1 | 83.0 |  |  |  |  |
|  | T2 | 68.2 |  |  |  |  |
|  | T3 | 42.9 |  |  |  |  |
|  | T4 | 0.0 |  |  |  |  |
| N stage |  |  | 4.161 (2.67- 6.49) | <0.001 | 2.185 (1.20- 3.99) | **0.011** |
|  | N0 | 76.9 |  |  |  |  |
|  | N1 | 35.1 |  |  |  |  |
| NLR |  |  | 1.522 (0.97- 2.40) | 0.070 |  |  |
|  | ≤1.78 | 68.7 |  |  |  |  |
|  | >1.78 | 57.9 |  |  |  |  |
| PLR |  |  | 1.351 (0.86- 2.12) | 0.192 |  |  |
|  | ≤192.0 | 65.7 |  |  |  |  |
|  | >192.0 | 56.9 |  |  |  |  |
| SII |  |  | 1.483 (0.95- 2.32) | 0.085 |  |  |
|  | ≤780 | 67.6 |  |  |  |  |
|  | >780 | 53.0 |  |  |  |  |

NA; not achieved, CEA; carcinoembryonic antigen, CA-19-9; carbohydrate antigen-19-9, NLR; neutrophil-to-lymphocyte ratio, PLR; platelet-to-neutrophil ratio, SII; systemic inflammatory index
