# Supplementary material for: Survival Outcomes According to Adjuvant Treatment and Prognostic Factors Including Host Immune Markers in Patients with Curatively Resected Ampulla of Vater Cancer
Source: PLoS One. 2016 Mar 14;11(3):e0151406. doi: 10.1371/journal.pone.0151406 (PMC4790941; doi:10.1371/journal.pone.0151406)
Supplement: S2 Table — (DOCX) [file pone.0151406.s003.docx]

**S2. The patterns of adjuvant treatment**

|  | **No. of all**  **patients** | **Total No. of patients with adjuvant treatment (%)** | **No. of patients with Radiotherapy** | **No. of patients with**  **Chemotherapy** | **No. of patients with**  **CCRT** | **No. of patients with**  **CCRT + maintenance chemotherapy** |
| --- | --- | --- | --- | --- | --- | --- |
| T1 | 68 | 10 (14.7) | 0 | 2 | 4 | 4 |
| T2 | 77 | 40(51.9) | 3 | 5 | 12 | 20 |
| T3 | 77 | 49(63.6) | 1 | 1 | 13 | 34 |
| T4 | 5 | 5(100) | 1 | 1 | 2 | 1 |
| N0 | 150 | 52(34.7) | 3 | 7 | 15 | 27 |
| N1 | 77 | 52(67.5) | 2 | 2 | 16 | 32 |
| Stage IA | 59 | 4(6.8) | 0 | 1 | 1 | 2 |
| Stage IB | 52 | 21(40.4) | 3 | 5 | 6 | 7 |
| Stage IIA | 40 | 28(70) | 0 | 0 | 8 | 20 |
| Stage IIB | 71 | 46(64.8) | 1 | 2 | 14 | 29 |
| Stage III | 5 | 5(100) | 1 | 1 | 2 | 1 |
| **Total** | **227** | **104(45.8)** | **5** | **8** | **32** | **59** |

CCRT; concurrent chemoradiotherapy
